# *Neisseria meningitidis* Serogroup W, Burkina Faso, 2012

**DOI:** 10.3201/eid2003.131407

**Published:** 2014-03

**Authors:** Jessica R. MacNeil, Isaïe Medah, Daouda Koussoubé, Ryan T. Novak, Amanda C. Cohn, Fabien V.K. Diomandé, Denis Yelbeogo, Jean Ludovic Kambou, Tiga F. Tarbangdo, Rasmata Ouédraogo-Traoré, Lassana Sangaré, Cynthia Hatcher, Jeni Vuong, Leonard W. Mayer, Mamoudou H. Djingarey, Thomas A. Clark, Nancy E. Messonnier

**Affiliations:** Centers for Disease Control and Prevention, Atlanta, Georgia, USA (J.R. MacNeil, R.T. Novak, A.C. Cohn, F.V.K. Diomandé, C. Hatcher, J. Vuong, L.W. Mayer, T.A. Clark, N.E. Messonnier);; Ministère de la Santé, Ouagadougou, Burkina Faso (I. Medah, D. Koussoubé, D. Yelbeogo, J.L. Kambou, T.F. Tarbangdo);; WHO Intercountry Support Team for West Africa, Ouagadougou (F.V.K. Diomandé, M.H. Djingarey);; Centre Hospitalier Universitaire Pédiatrique Charles de Gaulle, Ouagadougou (R. Ouédraogo-Traoré);; Centre Hospitalier Universitaire Yalgado, Ouagadougou (L. Sangaré)

**Keywords:** meningococcal meningitis, serogroup W meningococcal meningitis, Burkina Faso, serogroup A meningococcal conjugate vaccine, bacteria, children

## Abstract

In 2010, Burkina Faso became the first country to introduce meningococcal serogroup A conjugate vaccine (PsA-TT). During 2012, Burkina Faso reported increases in *Neisseria meningitidis* serogroup W, raising questions about whether these cases were a natural increase in disease or resulted from serogroup replacement after PsA-TT introduction. We analyzed national surveillance data to describe the epidemiology of serogroup W and genotyped 61 serogroup W isolates. In 2012, a total of 5,807 meningitis cases were reported through enhanced surveillance, of which 2,353 (41%) were laboratory confirmed. The predominant organism identified was *N. meningitidis* serogroup W (62%), and all serogroup W isolates characterized belonged to clonal complex 11. Although additional years of data are needed before we can understand the epidemiology of serogroup W after PsA–TT introduction, these data suggest that serogroup W will remain a major cause of sporadic disease and has epidemic potential, underscoring the need to maintain high-quality case-based meningitis surveillance after PsA–TT introduction.

In Burkina Faso, which lies within the meningitis belt of sub-Saharan Africa, rates of endemic meningitis are high, seasonal epidemics occur each year, and explosive epidemics occur every 5–12 years (*1*,*2*). Historically, these epidemics have been caused primarily by *Neisseria meningitidis* serogroup A (*3*,*4*). During late 2010, Burkina Faso became the first country to introduce a novel meningococcal serogroup A polysaccharide–tetanus toxoid conjugate vaccine (PsA-TT, MenAfriVac (Serum Institute of India Ltd, Pune, India). More than 11 million persons were vaccinated during ≈10 days in the eligible population of persons aged 1–29 years (*4*,*5*). Early evidence suggests that this aggressive strategy substantially reduced the rate of meningitis among persons in these age groups and in the general population because of high coverage and herd immunity (*5*). Since 2010, nine countries have implemented PsA-TT; the 100 millionth dose was given in December 2012.

Although *N. meningitidis* serogroup A has virtually disappeared in countries that have implemented nationwide vaccination campaigns, other serogroups, including *N. meningitidis* serogroup W and serogroup X, have the potential to cause epidemics. Sporadic disease and localized epidemics caused by serogroup X have been well described, but the potential of serogroup X to cause large epidemics remains unclear (*6*). During 2000 and 2001, serogroup W was associated with outbreaks in pilgrims to the Hajj and was followed by several clusters of cases worldwide (*7*–*9*). The largest reported outbreak caused by serogroup W occurred in Burkina Faso in 2002 and comprised ≈13,000 suspected cases (*10*). Sporadic cases and smaller outbreaks caused by serogroup W have continued to occur and have been described as occurring in several meningitis belt countries, including Burkina Faso (*11*–*15*). During 2012, Burkina Faso reported an increase in cases of serogroup W, raising questions about whether the increase resulted from a natural increase in disease or resulted from serogroup replacement after PsA-TT introduction. We analyzed national surveillance data and isolates from Burkina Faso to describe the epidemiology of serogroup W during 2012 and to assess changes and trends in serogroup W epidemiology around PsA-TT introduction.

## Methods

Burkina Faso has 2 complementary systems of population-based meningitis surveillance (*5*). Surveillance for reportable diseases is conducted by the Télégramme Lettre Official Hebdomadaire (TLOH), to which district-level aggregate reports of clinically defined meningitis cases and meningitis-related deaths are transmitted weekly. Enhanced surveillance for Maladies à Potentiel Epidémique (MPE) collects case-level demographic information and results of cerebrospinal fluid (CSF) examination and laboratory testing. For this analysis, we used data from TLOH for 2007–2012 and MPE enhanced surveillance data for 2010–2012. All 63 districts reported suspected meningitis cases to both surveillance systems during 2010–2012. Aggregate laboratory testing results for the 2007–2009 meningitis seasons and enhanced surveillance data for the 2002 meningitis season also were analyzed.

We used a modified case definition to classify cases. Suspected cases of meningitis were defined as sudden onset of fever with a stiff neck or, in infants, a bulging fontanelle. Probable bacterial meningitis was defined as a suspected case for which gram-stained CSF was positive (gram-negative diplococci, gram-positive diplococci, or gram-negative bacilli); probable meningococcal meningitis cases are limited to those with gram-negative diplococci. A confirmed case of meningitis was defined as a suspected or probable case for which *N. meningitidis*, *Haemophilus influenzae* type b or *Streptococcus pneumoniae* was isolated in culture from blood or CSF, or antigen was detected in CSF by latex agglutination or PCR. Latex agglutination–positive cases that were later negative by PCR or culture were reclassified as suspected cases.

Beginning in 2010, real-time PCR capacity was implemented at the national reference laboratory, and detection of *N. meningitidis*, *H. influenzae* type b, or *S. pneumoniae* genetic material by PCR was deemed confirmatory (*16*,*17*). Serogroup was determined by antigen detection or by PCR, with PCR deemed definitive. Because serogroup Y is seen infrequently as a cause of disease in Burkina Faso, all specimens positive for serogroup Y/serogroup W by latex agglutination and not tested by PCR or culture were assumed to be serogroup W.

The meningitis season in Burkina Faso is limited to epidemiologic weeks 1–24. The pre–PsA-TT implementation period was defined as January 1, 2007–December 31, 2010, and the post–PsA-TT implementation period as January 1, 2011–December 31, 2012. Incidence rates were cumulative yearly incidence per 100,000 population and were calculated with national, regional, and district population estimates from the Institut National de la Statistique et de la Démographie. Districts crossed the World Health Organization weekly epidemic threshold when the weekly incidence rate exceeded 10 suspected cases per 100,000 population (*18*).

Multilocus sequence typing, porin A (PorA), and ferric enterobacin transport (FetA) genotyping was performed on 61 serogroup W isolates (6 from 2011 and 55 from 2012). Multilocus sequence typing was performed by sequencing the internal fragment of 7 housekeeping genes (*19*). PorA and FetA genotyping was performed by sequencing the variable regions (VRs) of *porA* (VR1 and VR2) and the VR of *fetA*. All generated sequencing data were analyzed by using the Web-based software MGIP (http://mgip.biology.gatech.edu/home.php). Molecular types (sequence type [ST], clonal complex [cc], PorA, and FetA) were determined by searching the PubMLST database (http://pubmlst.org). Strain genotype was defined as ST(cc):PorA:FetA.

## Results

**Figure 1:**
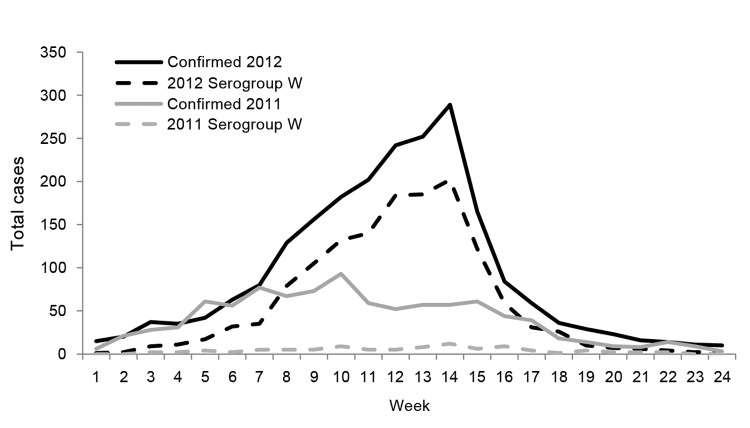
Laboratory-confirmed bacterial meningitis cases and *Neisseria meningitidis* serogroup W cases, Burkina Faso, 2011 and 2012 meningitis seasons. Data source: Maladies à Potentiel Epidémique (case-based surveillance).

During January 1–December 31, 2012, a total of 7,022 suspected meningitis cases (739 [11%] fatal) were reported through TLOH in Burkina Faso, corresponding to an incidence of 42.0 cases per 100,000 population. A total of 5,807 cases were reported through MPE case-based meningitis surveillance; 2,353 (41%) were confirmed, 415 (7%) were probable, and 3,039 (52%) were suspected meningitis cases. Of the 2,353 confirmed cases, 591 (25%) were confirmed by culture, 1,741 (74%) by PCR, and 21 (<1%) by latex agglutination. The predominant organism identified was *N. meningitidis* serogroup W (Table 1). More meningitis cases were reported during the 2012 than the 2011 meningitis season, and a higher proportion of cases were caused by serogroup W in 2012 than in 2011 (Figure 1).

**Table 1:** Laboratory-confirmed bacterial meningitis cases, Burkina Faso, 2012*

Bacterium, serogroup	Culture or PCR	Latex agglutination†	Total
*Neisseria meningitidis*			
A	0	0	0
W	1,438	13	1,451
X	207	NA	207
Y	4	0	4
C	1	0	1
Indeterminate	125	0	125
*Streptococcus pneumoniae*	527	7	534
*Haemophilus influenzae* type b	30	1	31
Total	2,332	21	2,353

*Data are from Maladies à Potentiel Epidémique case-based surveillance. NA, not applicable. †Excludes 62 latex agglutination–positive cases that were later ruled out by PCR and/or culture.

Of persons with *N. meningitidis* serogroup W infection during 2012, a total of 11% were <1 year of age, 29% were 1–4 years, 29% were 5–9 years, 16% were 10–14 years, and 15% were >15 years. This distribution is similar to that for serogroup W cases during the 2002 serogroup W epidemic, according to enhanced surveillance data (serogroup W: 16% persons were <1 year of age, 28% were1–4 years, 31% were 5–9 years, 8% were 10–14 years, and 17% were >15 years). The incidence of laboratory-confirmed serogroup W was 8.7 cases per 100,000 population in 2012, compared with 0.7 per 100,000 in 2011. During 2007–2010, when <15% of cases were laboratory confirmed, the incidence of serogroup W was <0.1 cases per 100,000 population (Table 2).

**Table 2:** Bacterial meningitis cases and incidence,Burkina Faso, 2007–2012*

Variable	Prevaccine					Postvaccine	
	2007	2008	2009	2010		2011	2012
Total no. cases reported in MPE	NA	NA	NA	3,413		3,415	5,807
Total no. specimens tested in aggregated laboratory results	550	93	241	NA		NA	NA
Confirmed or probable cases, no.	286	52	139†	1,408		1,306	2,768
*Neisseria meningitidis*, no. cases (Incidence):	257 (1.8)	49 (0.3)	43 (0.3)	170 (1.1)		278 (1.7)	1,788 (10.7)
*N. meningitidis* serogroup W, no. cases (Incidence):	4 (<0.1)	0	4 (<0.1)	10 (<0.1)		113 (0.7)	1,451 (8.7)
Total cases reported in TLOH	25,695	10,345	4,878	6,837		3,878	7,022

*Incidence = cases per 100,000 population. MPE, Maladies à Potentiel Epidémique (case-based surveillance); NA, not available; TLOH, Télégramme Lettre Official Hebdomadaire (aggregate case counts). †Only latex agglutination results available.

**Figure 2:**
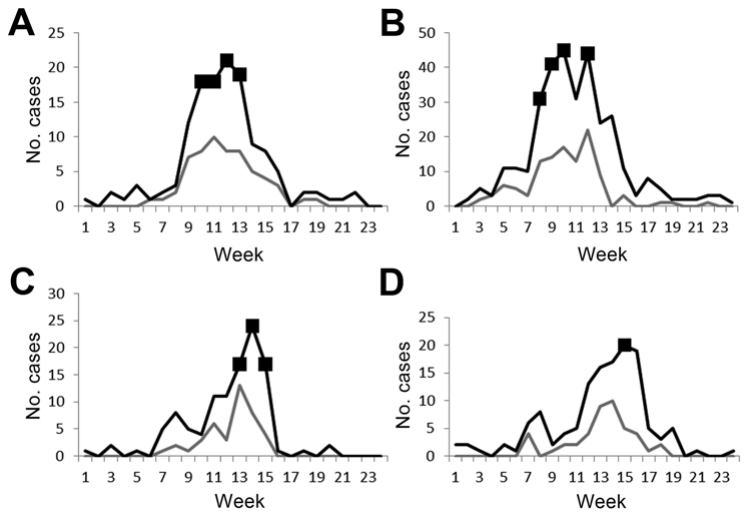
Total meningitis cases reported in Télégramme Lettre Official Hebdomadaire and laboratory-confirmed *Neisseria meningitidis* serogroup W cases for selected districts, Burkina Faso, 2012 meningitis season. A) District 1, population 150,000. B) District 2, population 300,000. C) District 3, population 100,000. D) District 4, population 200,000. Thin line indicates serogroup W cases; thick line, total cases; ▪ indicates weeks during which the number of cases crossed the weekly epidemic threshold. Data source: Télégramme Lettre Official Hebdomadaire and Maladies à Potentiel Epidémique.

The incidence of serogroup W varied by district, from 0.0 to 40.7 cases per 100,000 population (median 6.1/100,000) in 2012. By week 24, thirteen districts had crossed the weekly epidemic threshold of 10 suspected cases per 100,000 population (selected districts, Figure 2); *N. meningitidis* serogroup W was the predominant serogroup identified in 12 of these 13 districts (*N. meningitidis* serogroup X was the predominant organism identified in the 13th district). In no district did the cumulative incidence rate reach >100 cases per 100,000 population, the established definition of an epidemic, during 2012 (*20*). Three districts had reactive vaccination campaigns that used quadrivalent (A, C, W, Y) polysaccharide vaccine; in 2 of these districts, the number of cases crossed the weekly epidemic threshold. These districts were 2 of the last to cross the weekly epidemic threshold in 2012 during weeks 14 and 15. Cumulative incidence of serogroup W cases in these districts was 11.0 and 23.2 cases per 100,000 population, respectively.

We characterized 61 serogroup W isolates: 6 from 2011 and 55 from 2012, all belonging to cc11. The predominant genotype detected was ST-11(cc11):P1.5,2:F1–1, which accounted for 90% (55/61) of the total isolates. The remaining isolates differed only in the FetA and ST (ST-11[cc11]:P1.5,2:F6–3, 2/61 [3%]; ST-2961[cc11]:P1.5,2:F1–1, 2/61 [3%)]; and ST-9766[cc11]:P1.5,2:F1–1, 2/61 [3%]. Three STs were detected: ST-11 (57/61 [93%]), ST-2961 (2/61 [3%]), and ST-9766 (2/61 [3%]).

## Discussion

In 2012, the second year after PsA-TT introduction in Burkina Faso, the continued absence of serogroup A demonstrated the effect of this enormously successful vaccination campaign. *N. meningitidis* serogroup W was the predominant organism causing meningitis during the 2012 epidemic season. However, rates of serogroup W disease were substantially lower in 2012 (8.7 cases/100,000 population [Table 2]) than during the 2002 epidemic, when attack rates were ≈100–250 cases per 100,000 population (*10*,*21*).

The serogroup W clone associated with the Hajj-related outbreak during 2000 (*22*) has been present and a persistent cause of sporadic disease in Burkina Faso since the 2002 epidemic (R. Ouédraogo-Traoré, pers. comm.). The increase in serogroup W during 2012 might represent a natural increase in disease associated with waning population immunity in the 10 years since the last epidemic. This hypothesis is supported by the high proportion of serogroup W infections among younger children and the characterization of most serogroup W strains from 2012 as ST-11(cc11):P1.5,2:F1–1, the same genotype as the clone that caused the 2002 epidemic (*11*,*22*).

In Burkina Faso, rates of nasopharyngeal carriage of serogroup W are low (*23*). A previous study from Burkina Faso during the 2002 serogroup W epidemic demonstrated that serogroup W carriage infrequently induces protective immunity and that natural immunity against serogroup W may be lost (*24*). Serogroup replacement after nationwide vaccination with PsA-TT cannot be ruled out, however, and additional years of surveillance data, as well as nasopharyngeal carriage surveys, are required for a full understanding of whether serogroup replacement has resulted from PsA-TT vaccination. Preliminary data indicate that rates of serogroup W disease were lower during the 2013 meningitis season than during the 2012 meningitis season.

*N. meningitidis* has multiple genetic mechanisms to alter its antigenic profile, including capsular switching. Capsular switching has been observed among pneumococcal serotypes after the 7-valent pneumococcal conjugate vaccine was introduced in the United States, although the substantial declines in incidence of pneumococcal disease were maintained overall (*25*). Immediately after implementation of serogroup C meningococcal conjugate vaccine in the United Kingdom, no evidence of capsular replacement was found (*26*). Low rates of serogroup C disease in the United Kingdom have persisted, but serogroup Y has increased during the past several years in the United Kingdom and several other European countries (*27*). However, the effects of vaccination often are difficult to disentangle from natural fluctuations in meningococcal incidence and serogroup distribution.

With the recent shift in the epidemiology of bacterial meningitis in Burkina Faso after PsA-TT introduction, the current epidemic thresholds for predicting meningitis epidemics should be reevaluated. Although both serogroup W and serogroup X are known to cause epidemics, neither has been observed to cause the explosive epidemics of serogroup A or to have the periodicity observed for serogroup A epidemics. These thresholds were developed when the effects of disease were substantially higher so that epidemics could be anticipated and response activities (i.e., reactive vaccination campaigns) would prevent additional cases (*18*). In the 13 districts in Burkina Faso where meningitis reached the weekly epidemic threshold during 2012, the peak of reported cases was detected (Figure 2), but in no district was the cumulative annual epidemic threshold of 100 cases per 100,000 population met (*20*). During the 2012 meningitis season, 3 districts in Burkina Faso had vaccination campaigns that used quadrivalent polysaccharide vaccine. These are the first campaigns to use quadrivalent polysaccharide vaccine after PsA-TT vaccine; how many additional serogroup W cases were prevented and how administration of polysaccharide vaccination after PsA-TT will affect duration of protection against serogroup A disease are unknown.

The increases in serogroup W disease during 2012 were recognized because of the investment in high-quality meningitis surveillance in Burkina Faso, which underscores the importance of maintaining high-quality case-based meningitis surveillance capacity across the meningitis belt after PsA-TT introduction. Although additional years of data are needed to understand the epidemiology of meningococcal disease after PsA-TT introduction, it is evident that *N. meningitidis* will continue to cause sporadic disease and has potential to cause epidemics. In the meantime, countries need to effectively communicate the need for persons to remain diligent about seeking care for meningitis symptoms while not undermining public trust in the effectiveness of PsA-TT. The only available control strategy for serogroup W epidemics is reactive campaigns with trivalent or quadrivalent meningococcal polysaccharide vaccine. An affordable, multivalent vaccine, developed for use in the meningitis belt is needed in this disproportionately affected region to protect against epidemic meningitis.
